# Supplementary material for: A set of multi-entry identification keys to African frugivorous flies (Diptera, Tephritidae)
Source: Zookeys. 2014 Jul 24;(428):97–108. doi: 10.3897/zookeys.428.7366 (PMC4143993; doi:10.3897/zookeys.428.7366)
Supplement: Supplementary material 4 — Key to Capparimyia [file zookeys-428-097-s004.zip › SF4_ZooKeys_key to Capparimyia/lucid_player/help/help/get_score.htm]

Getting an Entities score value


# Getting an Entities score value

The Get Score function is useful for obtaining a quick view of how an
entity in the current Lucid Key has been scored and how or if it relates
to the entity you are attempting to identify.

To get the score information select the desired Feature/state and entity
and click the Get Score button located on the main toolbar.

A get score dialog will then show the scoring information on the selected
Entity for the given Feature or selected state.

**Note:** The Get Score
function availability is set by the key author and may not be available
in all keys.
